# Advances in Cerebellar TMS Therapy: An Updated Systematic Review on Multi-Session Interventions

**DOI:** 10.3390/biomedicines13071578

**Published:** 2025-06-27

**Authors:** Andrea Ciricugno, Sonia Paternò, Nicole Barbati, Renato Borgatti, Zaira Cattaneo, Chiara Ferrari

**Affiliations:** 1 IRCCS Mondino Foundation, 27100 Pavia, Italy; andrea.ciricugno@mondino.it (A.C.); renato.borgatti@mondino.it (R.B.); chiara.ferrari@unipv.it (C.F.); 2 Department of Brain and Behavioral Science, University of Pavia, 27100 Pavia, Italy; sonia.paterno01@universitadipavia.it; 3 Department of Humanities, University of Pavia, 27100 Pavia, Italy; 4 Department of Human and Social Sciences, University of Bergamo, 24129 Bergamo, Italy; nicole.barbati01@universitadipavia.it

**Keywords:** cerebellum, transcranial magnetic stimulation, rehabilitation, movement disorders, psychiatric disorders

## Abstract

**Introduction:** Cerebellar transcranial magnetic stimulation (TMS) has emerged as a promising neuromodulatory intervention for addressing motor, cognitive, and socio-affective deficits across a range of clinical populations. **Materials and Methods:** This systematic review aimed to synthesize recent evidence (2015–2025) on the efficacy, safety, and methodological characteristics of multi-session cerebellar TMS protocols used in rehabilitation settings. Following PRISMA guidelines, a comprehensive search of PubMed and Scopus was conducted to identify peer-reviewed studies applying multi-session cerebellar TMS in clinical populations for motor, cognitive, or affective rehabilitation. A total of 1750 records were screened, and 46 studies met the inclusion criteria. Data extraction included sample characteristics, study design, TMS protocol, targeted symptoms, outcomes, and risk of bias. **Results:** The results show that repeated sessions of cerebellar TMS are safe, well-tolerated, and associated with functional improvements primarily in motor disorders—such as spinocerebellar ataxia, Parkinson’s disease, multiple system atrophy, essential tremor, and post-stroke deficits—as well as in psychiatric populations, particularly patients with schizophrenia. **Discussion:** Evidence regarding the effects of cerebellar TMS on cognitive functions remains limited, though promising. Despite overall positive findings, the literature is limited by variability in stimulation parameters, protocol designs, and outcome measures, small sample sizes and potential publication bias. **Conclusions:** The review highlights the need for further large-scale and well-controlled trials to refine stimulation protocols, explore long-term effects, and clarify the underlying mechanisms of cerebellar TMS across motor, cognitive, and affective domains. This systematic review has been registered on PROSPERO (registration number: CRD420251067308).

## 1. Introduction

The cerebellum has long been recognized as a key structure for motor control, traditionally linked to the coordination of movement, balance, and posture. However, accumulating evidence over the past two decades has expanded this view, highlighting its crucial involvement in higher-order cognitive and socio-affective functions [1,2]. Cerebellar abnormalities have been associated not only with motor dysfunctions, but also with impairments in executive functions, working memory, attention, language, emotional regulation, and social cognition [3]. These insights are supported by clinical studies in individuals with focal cerebellar lesions (e.g., stroke) [4] or cerebellar degeneration (e.g., hereditary ataxias) (for a review, see [5]), who often present with overlapping cognitive and socio-affective deficits. Moreover, cerebellar structural and functional alterations have been reported across various neurological and psychiatric conditions, including essential tremor, Parkinson’s disease (PD), multiple sclerosis, Alzheimer’s disease (AD), schizophrenia, and mood disorders [6,7]. A prevailing hypothesis suggests that these diverse symptoms may stem from disruptions in cerebellar modulation of cerebro-cortical networks, pointing to a transdiagnostic role of the cerebellum in brain function and dysfunction (see [8]).

Among non-pharmacological interventions, transcranial magnetic stimulation (TMS) targeting various cortical regions has undergone a marked expansion in recent years, with mounting evidence supporting its efficacy across a wide range of neurological and psychiatric conditions [9,10]. For example, repetitive TMS (rTMS) applied over the motor cortex has been used as a valuable rehabilitative tool to improve motor function in patients with stroke, traumatic brain injury (TBI), or neurodegenerative diseases such as PD and multiple sclerosis [11]. Beyond motor rehabilitation, rTMS targeting prefrontal, temporal, or parietal cortices has shown promising results in enhancing cognitive function in both acute (e.g., stroke) and degenerative (e.g., AD) conditions, particularly when paired with cognitive training [12]. Notably, rTMS can be delivered either alone or in conjunction with conventional rehabilitation programs, with evidence suggesting that repeated sessions can boost and accelerate treatment responses. The therapeutic use of TMS in psychiatry has also experienced substantial growth. Repeated prefrontal stimulation has proven effective in reducing negative symptoms and auditory hallucinations in schizophrenia [13,14], as well as in improving mood in patients with treatment-resistant depression [15]. These findings support the role of TMS as a neuromodulatory technique capable of modulating dysfunctional brain circuits and promoting neural plasticity, thereby supporting recovery of motor, cognitive, and affective functions. When applied repeatedly, TMS may be effectively integrated into conventional neurorehabilitation protocols to amplify therapeutic outcomes, especially in patients with neurodegenerative, cerebrovascular, and psychiatric conditions. Its non-invasive nature and favorable safety profile further reinforce its translational value, particularly for vulnerable clinical populations. Reflecting its clinical relevance, since 2008 the U.S. Food and Drug Administration (FDA) has approved TMS as a valuable treatment tool; after that several TMS protocol have been approved by the FDA and European regulatory agencies, including those for treatment-resistant depression, obsessive–compulsive disorder (OCD), chronic pain, and smoking cessation [16].

When applied to the cerebellum, TMS has emerged as a powerful experimental tool for investigating the causal contribution of this structure to motor, cognitive, and socio-affective functions in healthy individuals [2,17,18]. Beyond its use in basic research, a growing body of clinical studies indicates that repeated cerebellar TMS sessions are safe, well-tolerated, and may represent a promising therapeutic strategy for addressing motor dysfunctions (e.g., [19]), cognitive impairment (e.g., [20]), and psychiatric symptoms (e.g., [21]). These findings position cerebellar TMS as a versatile and potentially transdiagnostic neuromodulatory intervention. Despite the increasing interest in cerebellar TMS, the current literature lacks a comprehensive and up-to-date synthesis of its clinical applicability. In this context, a systematic review evaluating the efficacy, safety, and tolerability of repeated cerebellar TMS across various diagnostic categories is critical to fully elucidate its therapeutic potential and guide its implementation in neurorehabilitation protocols.

In this systematic review, we provide a critical overview of studies from the past decade investigating the effects of repeated cerebellar TMS on motor, cognitive, and psychiatric symptoms in clinical populations. We begin with a brief overview of cerebellar anatomy and functional neurophysiology, emphasizing its role across motor, cognitive, and affective domains, as well as the consequences of its dysfunction. We then examine key methodological and translational aspects of cerebellar TMS application and conclude with future directions aimed at optimizing its use within rehabilitative and clinical frameworks.

### Cerebellar Anatomy and Functions

The cerebellum exhibits a well-established functional topography, reflecting its involvement in a diverse range of motor and non-motor processes. Anterior regions (lobules I–V and VIII) are primarily associated with motor control, while posterior lobules (VI and VII) are selectively recruited during cognitive tasks, and lobules IX and X are implicated in vestibular processes [22,23,24]. This functional dissociation is supported by lesion studies, with damage to anterior lobules typically resulting in motor deficits such as ataxia. In contrast, lesions in the posterior cerebellum are linked to the cerebellar cognitive affective syndrome (CCAS), characterized by impairments in executive function, visuospatial abilities, language, and affect regulation [3,25].

Furthermore, an even finer functional specialization emerges within the posterior cerebellum. For instance, bilateral Crus I and II (in lobule VII) are predominantly involved in executive functions and working memory. Regions supporting language are lateralized in the right posterior cerebellum, while those supporting visuospatial processing are lateralized in the left. Medial lobule VII contributes to affective processes [26,27,28]. Moreover, recent TMS findings further reveal that the posterior cerebellum involvement in socio-cognitive processes reflects a medial-to-lateral gradient, with medial regions engaged in basic emotional processing, and lateral areas supporting higher-level social cognitive operations [29].

The topographical organization of the cerebellum reflects the connectivity pattern between the cerebellum and cerebrum. The cerebellum is interconnected with the cerebrum via two primary polysynaptic closed-loop circuits: the cortico-ponto-cerebellar pathway, which conveys cortical input to the cerebellum, and the cerebello-thalamo-cortical pathway, which forwards information from the cerebellum to the cerebral cortex [30]. Evidence from both animal models and human studies demonstrates that anterior cerebellar regions form loops with primary motor cortices, while posterior regions are functionally linked to associative cortical areas [31,32]. Still, functional asymmetries in cerebral association networks are paralleled in the cerebellum, with individuals showing stronger cerebral lateralization also exhibiting stronger cerebellar asymmetries [27,33,34]. This asymmetry reinforces the notion of homotopic mapping between cortical and cerebellar regions, particularly within associative networks.

The cerebellar cortex exhibits a relatively uniform anatomy and physiology, which has given rise to the idea that this structure performs the same computational function across motor, cognitive, and affective domains [35]. Schmahmann [36] introduced the concept of Universal Cerebellar Transform, proposing that the cerebellum applies the same computation—such as prediction and error correction—across functions. Similarly, the internal model hypothesis posits that the cerebellum generates predictive models not only for motor control but also for cognitive tasks, such as working memory, language, and social cognition [24,37,38,39]. Yet, more recently, the notion of cerebellar “multiple functionalities” has been proposed, suggesting that despite its uniform circuitry, the cerebellum may support qualitatively distinct computations depending on the context or network in which it is embedded [40,41]. While several leading hypotheses have been proposed, the precise nature of the cerebellum’s computational role across domains remains an open question. Future studies should aim to determine whether the cerebellum serves as a domain-general predictive engine or as a dynamic processor flexibly adapting its computations to the specific task or network—a distinction with profound implications for both theoretical models and clinical applications.

## 2. Materials and Methods

This review was conducted according to the framework proposed by Peters et al. [42] for scoping studies.

### 2.1. Identifying the Review Questions

The research question was: “What evidence in the available literature supports cerebellar TMS as a rehabilitative treatment for motor, cognitive and affective deficits?”. The present review aims to (i) map and summarize the evidence from the last 10 years on the rehabilitative effects of cerebellar TMS on motor, cognitive, and affective symptoms, and (ii) provide critical considerations on potential implications for neurological and psychiatric disorders.

### 2.2. Inclusion Criteria

The criteria for study inclusion were as follows: (i) usage of multiple TMS sessions targeting the cerebellum in clinical populations for (ii) the rehabilitation of motor and/or cognitive and/or affective symptoms, as assessed by performance-based measures, questionnaires, or qualitative outcomes (e.g., verbal report), reported in (iii) research articles published in peer-reviewed English-language journals. Records considered as not pertinent were as follows: animal studies, studies on healthy individuals, single-case studies, studies using other stimulation tools (e.g., transcranial direct current stimulation, vagus nerve stimulation, deep brain stimulation), studies applying TMS to other brain areas, studies applying single-session TMS, neuroimaging studies, or studies using other investigative approaches such as pharmacological interventions. Additionally, documents other than primary research articles and study protocols (e.g., reviews, book chapters, commentaries) were excluded. At each stage of the selection process, if one or more exclusion criteria were identified in the title, abstract, or full text, the record was not selected for inclusion.

### 2.3. Search Strategy

A.C. and N.B. conducted the literature search, by screening scientific online databases (PubMed and Scopus) to identify pertinent studies, using the following text string: (cerebellum OR cerebellar) AND (brain stimulation OR neurostimulation OR transcranial magnetic stimulation) AND (patients) and (motor OR psychiatry OR symptoms OR cognition OR rehabilitation). The period considered for study inclusion was January 2015–May 2025. No other restrictions were imposed on the document type or language of the records.

### 2.4. Evidence Screening and Selection

**Figure 1:**
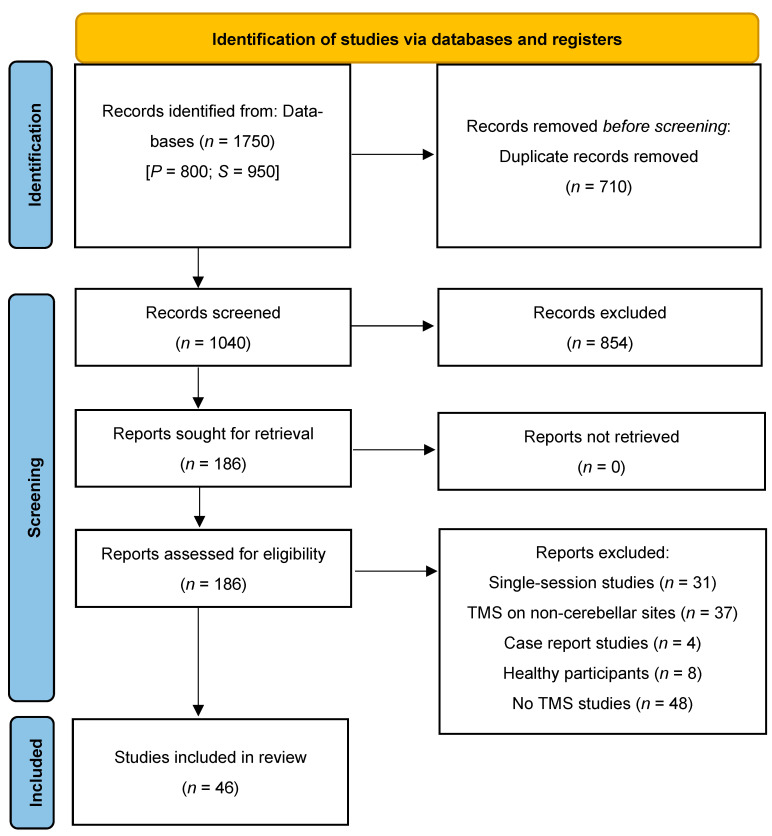
Flowchart of the study selection procedure. Abbreviations: P: Pubmed; S: Scopus.

After removing duplicates, A.C. and N.B. screened the titles of the records (title screening) identified by the search string and excluded those records that did not fit the topic of the review. Of the remaining records, they screened the abstract (abstract screening) and excluded those whose content was judged not to be relevant for the present review. If some methodological information could not be retrieved by screening the abstracts, the two authors read the full texts (text screening) to determine whether to include the record. The list of records, after duplicates were removed, was split into two parts; one reviewer screened the first half, and the other screened the second half. Figure 1 depicts the study selection procedure.

### 2.5. Data Extraction and Charting

Data were charted referring to the review question “What is the evidence, described in the published literature, of cerebellar transcranial magnetic stimulation as a rehabilitative treatment for motor, cognitive, and affective deficits?”. The list of selected records was divided into two parts. For each part, data were extracted by one reviewer and a 25% sample was checked for accuracy and completeness by the other [43].

Table 1 reports information regarding the methodological aspects of the selected studies: first author and year of publication; sample size; mean age; diagnosis; study design (i.e., within/between-subject, cross-over); whether the study was sham-controlled; had a healthy control group; applied a blinding procedure; included a follow-up to detect long-lasting effects of the stimulation; whether or not medication intake were reported or controlled for; outcome measures; main findings.

Table 2 reports the technical details of the TMS protocols: first author and year of publication; type of stimulation protocol (e.g., low/high frequency rTMS, iTBS, cTBS); target site(s); type of coil used; frequency and intensity of the stimulation; targeted cerebellar site; whether an MRI-guided navigation system was used; number of sessions; timing of the stimulation (online or offline); whether stimulation was combined with other treatments; whether information on safety, tolerability and adverse events were reported or not. 

### 2.6. Risk of Bias Assessment

The risk of bias of the RCTs included in the review was conducted using the revised Cochrane risk of bias tool (ROB V.2.0) [88]. The following domains were assessed: randomization process, deviations from intended interventions, missing outcome data, measurement of the outcome, and selection of the reported result. The risk of bias for each domain was graded as either low, high, or unclear, and then summarized into an overall judgment. A study was considered to have a low risk of bias only when all domains were graded as having a low risk of bias. A study was considered as having an unclear risk of bias if one domain was graded as having an unclear risk of bias and no other domains were graded as having a high risk of bias. A study was considered as having a high risk of bias if at least one domain was graded as high risk. The NHLBI (National Heart, Lung, and Blood Institute, 2014) Quality Assessment Tool to evaluate the methodological quality of non-RCTs studies with a before-and-after (pre-post) design with no control group. All studies were evaluated independently by three investigators (A.C., N.B., and S.P.); discrepancies between investigators were settled by consensus or by a panel discussion with C.F. and Z.C. 

## 3. Results

The first section describes the results of the research strategy and selection process, while the second section reports data synthesized in a descriptive format, to identify different aspects of the literature as outlined in the key question. Stimulation technical details, methodological choices, and outcome characteristics were reported.

### 3.1. Study Selection for Review

The literature search identified a total of 1750 records: 800 from Pubmed and 950 from Scopus. After removing duplicates (*n* = 710), the titles of 1039 records were screened. Of these, 854 records were excluded as non-pertinent whenever the title was indicative of the presence of one or more of the exclusion criteria described above. The abstracts of the remaining records (*n* = 181) were screened against the inclusion/exclusion criteria. This stage of the selection process led to the identification of 64 potentially eligible studies. After reading the text of these records, 19 were further excluded. Thus, 46 records were deemed eligible and included in data extraction and charting (see Figure 1).

### 3.2. TMS Protocols

Out of the 46 records included in the data extraction and charting phase, 24 (54.3%) applied an intermitted theta-burst stimulation protocol, with frequencies ranging from 5 to 7 Hz, 11 (23.9%) applied a low-frequency (1 Hz) stimulation protocol, and 6 (13.1%) a high-frequency stimulation protocol, at frequencies from 5 to 10 Hz. The remaining 4 studies (8.7%) opted for a continuous theta-burst stimulation protocol. One of the studies employing cerebellar cTBS combined this stimulation with low-frequency stimulation of the primary motor cortex. Furthermore, one study employed a cerebellar–motor cortex paired associative stimulation protocol delivering 120 pairs of pulses at 0.2 Hz. 30 studies (65.2%) targeted different cerebellar sites (i.e., vermis, left and right cerebellum) within the same session, first on one site and then subsequently on the others, with short breaks between each site. The remaining 16 studies (34.8%) targeted a single cerebellar site: the vermis was targeted in 7 studies, the contralesional lateral cerebellum in 6 studies, the ipsilesional lateral cerebellum in 2 studies, and the left cerebellum in 1 study. Four studies also stimulated non-cerebellar regions. Cha et al. [51] used occipital cortex stimulation as a control condition. Li et al. [63] stimulated the primary motor cortex alone in one group of participants and applied a sequential stimulation of the motor cortex and cerebellum in another, similar to the approach used by Liu et al. [48]. Rosso et al. [65], instead, employed paired-pulse stimulation of the cerebellum and primary motor cortex. 

As for the intensity parameter, 18 studies (39.1%) stimulated at 80% of the participants’ motor threshold (MT), with 10 studies measuring it at rest (rMT) and 8 during active contraction (aMT). A total of 10 studies (21.7%) applied TMS at 90% of the rMT and 12 (26.1%) at 100% (9 using rMT, 2 aMT, and 1 study not specified). Four studies employed a fixed frequency of 50% of the maximum stimulator output (MSO), while one study delivered TMS at 100% of MSO (using a Magstim 200 stimulator with a 14 cm circular coil) [52]. One study employed individualized frequencies across participants using the maximum tolerated intensity level that would not elicit pain for each site [51].

Concerning the coil type and geometry, 30 (65.2%) studies used a figure-of-eight coil—likely stimulating more superficial, posterior regions of the cerebellum—10 (21.7%) studies used a double cone coil and 5 (10.9%) a circular coil. One study did not report any information about the coil type [81].

In 31 (67.4%) studies, patients received multiple sessions of cerebellar TMS treatment without any concomitant rehabilitative activity. In turn, in the other 15 studies, the stimulation was combined with conventional rehabilitation training, including physiotherapy, swallowing/dysphagia therapy, gaze stability training, and acupuncture. In 13 of these studies, cerebellar stimulation was delivered using an offline modality, meaning while patients were resting, before starting with routine physical/motor training. Only two studies [79,80] applied cerebellar TMS online, meaning while patients were engaged in rehabilitation activities (i.e., speech and physiotherapy). The minimum number of repeated stimulation sessions was five, as reported in six studies (13.0%). Overall, 34 studies (73.9%) administered between 10 and 15 sessions, while 6 studies applied 20 or more sessions, with one study reaching up to 48 stimulation sessions.

Although neuronavigated TMS on individual magnetic resonance images (MRI) scans is encouraged to enable precise targeting and decrease interindividual variability [89], only 10 studies complied with this requirement. In the remaining 36 studies, the coil positioning was based on anatomical landmarks. 

Most of the studies (35, 76.1%) reported information on cerebellar TMS tolerability and correlated sensations, including transient headache, discomfort due to contraction of the neck muscles, discomfort over the stimulated area, fatigue, nausea, and vertigo. In one study [61], hypomania was reported as an adverse effect in two patients. In 12 studies, no adverse events were observed. 

### 3.3. Studies Methodology and Characteristics

This paragraph provides a descriptive overview of the methodological features, schematized in Table 2.

Out of the 46 TMS studies, 36 (78.2%) adopted a between-subjects design, in which patients received either real or sham stimulation. Among the remaining studies, five (10.9%) adopted a within-subjects design, with all the patients receiving real cerebellar TMS, and four (8.7%) a cross-over design, where patients received sham cerebellar TMS followed by real stimulation, or vice versa. One study [76] was conducted in two phases, using a between-subjects design in phase 1 and a within-subjects design in phase 2.

Overall, the majority of the studies (37, 80.4%) were sham-controlled. The sham condition was obtained by using one of the following methods: a modified coil, able to mimic the sound click and sensation of real TMS; setting the stimulation to a low intensity (10% or 40% of the individual MT); or tilting the coil to 90 degrees. 

As for the blinding procedure, 31 studies (67.4%) employed a double-blind procedure, while 8 studies adopted a single-blind procedure. No indication of the use of any blinding method was found in the remaining 7 studies. 

Regarding the inclusion of follow-up measures, they were detected in 27 studies examining the potential long-lasting therapeutic effects of cerebellar TMS.

In total, the TMS studies included in the review process tested 1503 patients. All patients were adults. Only five studies included a healthy control group. Most studies (21, 45.6%) enrolled patients diagnosed with movement disorders, including spinocerebellar ataxias, dystonia, Parkinson’s disease, essential tremor, and multiple system atrophy. In total, 14 studies (30.4%) enrolled post-stroke patients and those with associated symptoms, such as dizziness, dysphagia, limb hemiplegia/hemiparesis, and unilateral neglect. Critically, these studies did not include patients with cerebellar stroke (except one study that also enrolled some patients with cerebellar stroke), but primarily focused on patients with cortical or subcortical strokes within the middle cerebral artery territory, or strokes affecting the brainstem. Of the remaining 11 studies, 5 recruited patients with non-motor disorders, such as Alzheimer’s disease, drug-resistant epilepsy, multiple sclerosis, and Mal de Débarquement syndrome, while 6 enrolled psychiatric patients, all with a diagnosis of schizophrenia. 

The use of medications by the patients was either reported or controlled for in 25 studies. The remaining 21 did not report any information on current medication intake. 

Concerning the outcome measures, the review process shows that most studies (36, 78.3%) evaluated the effects of cerebellar TMS on motor functions, including, but not limited to, balance, ataxia severity, tremor, and gait by means of standardized scales and questionnaires. Six studies (13.0%) assessed affective outcomes, including measures of affective states and symptom severity in schizophrenic patients. Two studies employed a comprehensive set of scales to measure motor, cognitive, and affective outcomes, while only one study assessed stimulation effects on epilepsy. Furthermore, six studies (13.0%), in addition to using standard scales and questionnaires for motor, cognitive, and/or affective assessment, also included physiological measures such as resting-state EEG and fMRI.

Concerning the findings, ref. [42] (91.3%) studies reported that cerebellar TMS significantly improved at least one of the assessed outcomes. In turn, only four studies reported no significant effects of real TMS on either motor or affective measures. This could pave the way for a discussion of publication bias. Indeed, one may suspect that other studies with negative findings could not be published because they are less likely to be accepted by journals [90]. That said, except a few studies, significant cerebellar stimulation effects on motor, cognitive, and affective functions were found in all the TMS studies included in the review process.

### 3.4. Risk of Bias 

**Figure 2:**
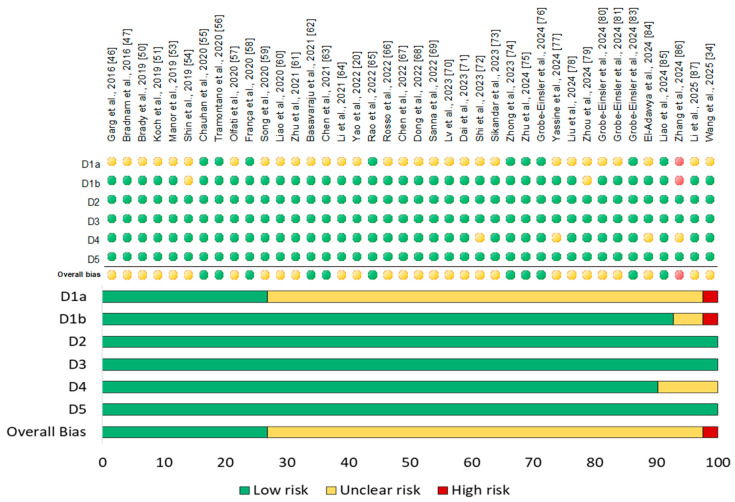
Risk of bias summary of all included RCTs (*n* = 40). D1a: Randomization process; D1b: timing of identification or recruitment of participants; D2: deviations from intended intervention; D3: missing outcome data; D4: measurement of the outcome; D5: selection of the reported result.

Among all studies, forty were RCTs and five were non-RCTs with a pre-post design and no control group. The Cochrane risk of bias (ROB) assessment for RCTs estimated that 11 studies (27.5%) were considered at low risk of bias and 28 studies (70%) were assessed as having an unclear risk of bias mainly due to the lack of allocation concealment, issues in participants’ timing of identification or recruitment, or lack of outcome assessors blinding, while 1 study showed a high risk of bias because of baseline imbalances that suggest differential identification/recruitment of individual participants between intervention groups. The risk of bias with respect to different domains– randomization, allocation, blinding, and outcome assessment—and reporting is summarized and represented in a graph as percentages below using RevMan 5.4 software (“RevMan 5.4”) (Figure 2). The quality assessment of non-RCTs, carried out using the NHLBI (National Heart, Lung, and Blood Institute, 2014) Quality Assessment Tool, found that one study had a good methodological quality; three studies were of fair quality, while one study had poor quality, mostly because of issues related to the small sample size, lack of blinding, and high rate of loss to follow-up after baseline (Table 3).

## 4. Discussion

The cerebellum plays a crucial role in motor control and coordination. However, growing evidence highlights its involvement—particularly its posterior regions—in high-level cognitive, social, and affective functions through bidirectional connections with cortical and subcortical areas of the cerebrum. Notably, cerebellar alterations can lead not only to sensorimotor deficits but also to a constellation of cognitive, social, and affective dysfunctions, collectively known as Cerebellar Cognitive Affective Syndrome (CCAS) [3].

Among non-pharmacological approaches, TMS, whether used alone or alongside other treatments, has proven effective in rehabilitating motor, cognitive, and socio-affective functions across various clinical conditions [11,12]. When applied to the cerebellum, it is a valuable tool for exploring the causal role of this region in healthy individuals (for review see, [2,17,18] and is considered a safe and well-tolerated method for relieving neurological and psychiatric symptoms [19,21,91]. Here, we identify and review the evidence from the past ten years on the effectiveness of multi-session cerebellar TMS as a rehabilitative treatment for various clinical conditions. Our goal is to provide an updated overview of this topic, assessing the current state of research and exploring future directions for advancement.

### 4.1. Movement Disorders

Given the traditional view of the cerebellum as primarily responsible for sensorimotor functions, it is not surprising that most of the reviewed studies focused on using cerebellar TMS to treat motor symptoms in patients with movement disorders. Among these conditions, spinocerebellar ataxia—particularly SCA3—was a frequent focus of investigation. In patients with spinocerebellar degeneration, repeated sessions of LF-rTMS and iTBS applied to one cerebellar hemisphere or multiple sites within the same session (e.g., vermis, left and right cerebellar hemispheres) led to significant improvements in ataxia severity [52,57,66,68,71,72,77]. Similar benefits, in the same population, were observed with HF-rTMS [78] and when iTBS was combined with conventional physiotherapy [79,80]. Interestingly, these clinical improvements following cerebellar stimulation were linked to increased motor cortical excitability [68] and heightened activity in distal brain regions, such as the temporal and occipital cortex [77]. In turn, in patients with cervical dystonia, repeated iTBS over the cerebellar hemispheres, combined with active motor control training, improved motor coordination and pain but did not affect motor cortical excitability or inhibition [46]. This highlights how cerebellar TMS may influence functionally connected brain networks, with variations depending on the specific neurological disorder.

Further evidence comes from studies on multiple system atrophy with predominant cerebellar dysfunction (MSA-C), where iTBS over the cerebellar hemispheres increased cerebello-frontal connectivity [58] and improved ataxia, balance, and fatigue [58,86]. Similarly, HF-rTMS targeting both the cerebellum and bilateral M1 enhanced motor control in MSA patients, with the degree of improvement correlating with increased resting-state complexity within the motor network [48].

The effects of cerebellar TMS on essential tremor or PD have also been explored, given the cerebellum’s role in a network of oscillators contributing to tremor generation (e.g., [92]). However, studies reported mixed results. Five days of iTBS applied to multiple cerebellar targets (i.e., vermis, left and right cerebellar hemispheres) effectively improved bradykinesia and gait in PD patients, with benefits lasting up to a month. However, the effects on tremor and postural stability were less consistent [75,82]. The duration of stimulation appears to be a critical factor when using LF-rTMS. While four weeks (five sessions per week) of stimulation over both cerebellar hemispheres significantly reduced essential tremor [81,83] and increased fronto-parietal connectivity, as measured by resting-state EEG [81], shorter protocols (5 or 10 days) failed to improve tremor or motor performance and did not modulate motor cortical excitability compared to sham stimulation [53,56] or treatment with propranolol [69].

Cerebellar TMS has also been explored as a therapeutic approach for motor symptoms in neurological conditions beyond primary movement disorders, notably in multiple sclerosis (MS). Importantly, approximately one-third of MS patients exhibit cerebellar involvement or disruption of cerebellar circuits, either during acute exacerbations or throughout chronic disease progression [93]. In this context, preliminary evidence has demonstrated that two weeks of bilateral iTBS over the lateral cerebellum, when combined with vestibular rehabilitation, can significantly improve gait, balance, and gaze stability in patients with advanced disability levels, compared to vestibular rehabilitation alone [55]. These findings were corroborated in a subsequent study involving individuals with relapsing-remitting MS, where a protocol of twelve iTBS sessions targeting multiple cerebellar sites (including the vermis and bilateral hemispheres) alongside balance and gait rehabilitation resulted in notable enhancements in walking ability [76]. Notably, these improvements were observed exclusively following active stimulation, with no comparable effects in the sham condition. Furthermore, diffusion tensor imaging revealed increased white matter integrity within both the cerebello-thalamo-cortical and the cortico-ponto-cerebellar pathways, suggesting a neuroanatomical substrate underpinning the observed functional gains. Interestingly, periodic repetition of the same stimulation protocol at three-month intervals appeared to consolidate both clinical outcomes and structural brain changes, highlighting the potential of cerebellar iTBS as a promising adjunctive strategy for sustained motor rehabilitation in MS.

Overall, these findings highlight the potential of cerebellar TMS as a therapeutic tool for movement disorders, with promising effects on ataxia, gait, and motor control. However, responses appear to vary across conditions, and factors such as stimulation protocol, duration, and disease-specific mechanisms likely play a crucial role in determining efficacy.

### 4.2. Post-Stroke Recovery

Motor dysfunction is one of the most common complications following stroke, affecting up to 80% of survivors and severely compromising patients’ independence and quality of life. Among the reviewed studies, thirteen examined cerebellar neurostimulation as a rehabilitative approach for various motor deficits following stroke, with most focusing on cortical or subcortical strokes within the middle cerebral artery territory or strokes affecting the brainstem. Notably, protocols involving ten or more sessions of cerebellar iTBS—administered either alone or in conjunction with conventional physiotherapy—demonstrated significant improvements in both upper and lower limb motor function. Stimulation of the contralesional or ipsilesional lateral cerebellum led to enhancements in gait, balance, and reductions in spasticity. Importantly, these improvements surpassed those observed with iTBS applied to the primary motor cortex [84], conventional physiotherapy alone [60], or sham stimulation [50,59,62]. These clinical benefits were accompanied by neurophysiological changes, including increased motor cortical excitability in the unaffected hemisphere [62] and enhanced theta-band activity over the posterior parietal cortex of the affected hemisphere [50]. Collectively, these findings suggest that cerebellar stimulation may promote long-term potentiation within the cerebellar cortex, thereby modulating activity across connected cortical networks.

Building upon this rationale, several studies have explored dual-site stimulation paradigms, combining cerebellar TBS with motor cortex stimulation to further potentiate rehabilitation outcomes. For example, Li et al. [63] administered LF-rTMS over the motor cortex prior to cerebellar TBS within each session, in combination with physiotherapy. This multimodal protocol yielded superior reductions in muscle spasticity and limb dyskinesia compared to stimulation of either site in isolation. Similarly, Rosso et al. [65] implemented a paired associative stimulation (PAS) protocol that integrated cerebellar and motor cortex stimulation alongside physiotherapy, resulting in significant improvements in hand dexterity (although not grip strength), accompanied by increased activation in the ipsilesional primary motor cortex, as evidenced by fMRI.

Further supporting the therapeutic potential of cerebellar stimulation, five sessions of iTBS targeting the cerebellar vermis with a double-cone coil markedly alleviated dizziness and resolved ipsilesional nystagmus in patients with lateral medullary infarction, with effects persisting for up to six months [47]. These outcomes are likely attributable to the stimulation of the nodulus and uvula, critical cerebellar structures involved in vestibular regulation, leading to improved modulation of the vestibulo-ocular reflex (VOR). Correspondingly, the authors reported increased regional cerebellum-to-brainstem cerebral blood flow ratios following stimulation [47].

Dysphagia, another frequent post-stroke complication, affects between 37% and 78% of patients [94] and is associated with heightened risks of dehydration, malnutrition, and mortality [95]. Across the reviewed literature, ten sessions of HF-rTMS delivered bilaterally to the cerebellar hemispheres—using either a circular or figure-of-eight coil—combined with conventional swallowing rehabilitation, significantly improved swallowing function relative to sham stimulation [67,73] and unilateral cerebellar stimulation [67]. Notably, these functional gains were accompanied by increased motor-evoked potentials (MEPs) recorded from the mylohyoid muscle [67]. Comparable outcomes were observed following two weeks of iTBS applied to the bilateral cerebellar hemispheres, either as a standalone intervention [70] or in combination with traditional dysphagia therapy [64], with benefits maintained at a four-week follow-up [64].

Beyond motor symptoms, stroke frequently results in cognitive impairments and mood disturbances, arising from both focal lesions and disrupted network connectivity, which together impede recovery and elevate the risk of recurrence and mortality. Among the reviewed studies, only two investigated the efficacy of cerebellar stimulation on cognitive and/or emotional domains in stroke patients [85,87]. In one study, ten sessions of iTBS applied to the contralesional lateral cerebellum, combined with conventional rehabilitation, produced greater improvements in global cognitive function (as assessed by the Montreal Cognitive Assessment and Mini-Mental State Examination), naming abilities, working memory, and mood compared to rehabilitation alone. Crucially, these clinical improvements were associated with modulations in resting-state activity within the salience and attentional networks [85]. In the study by Ye and colleagues [87], twenty sessions of iTBS targeting the contralesional cerebellum in patients with unilateral neglect led not only to clinical improvements in neglect symptoms but also to increased excitability of the ipsilesional parietal cortex and enhanced functional reorganization within frontoparietal networks.

Interestingly, none of the reviewed studies applied cerebellar TMS in patients with cerebellar strokes, with some explicitly excluding such cases as part of their study criteria. Only one study included patients with cerebellar stroke; however, the data were limited and not analyzed separately from those of patients with non-cerebellar stroke. This gap highlights an important avenue for future research, as the application of cerebellar stimulation in post-cerebellar stroke rehabilitation could provide valuable insights into its therapeutic potential for this specific population. Taken together, these findings underscore the promising role of cerebellar neurostimulation as a versatile and effective adjunct in the rehabilitation following non-cerebellar strokes (in particular of those affecting cortical and subcortical regions within the territory of the middle cerebral artery). By facilitating recovery across a spectrum of motor, vestibular, swallowing, cognitive, and emotional functions, cerebellar stimulation holds considerable potential for the development of more integrated and personalized therapeutic strategies in stroke care. 

### 4.3. Non-Motor Neurological Disorders

The application of cerebellar TMS to non-motor disorders remains relatively underexplored, with only three studies to date investigating clinical conditions not primarily defined by motor deficits. In the first study, Cha and colleagues [51] examined the effects of cerebellar TMS in patients with Mal de Débarquement Syndrome (MdDS), a motion perception disorder characterized by persistent oscillating vertigo following prolonged exposure to periodic motion, such as during extended travel by sea, air, or land [96]. Participants initially received a single session of cTBS over the occipital cortex, cerebellar vermis, and lateral cerebellar hemisphere, and were asked to report acute changes in vertigo severity. Subsequently, they underwent 10 to 12 sessions targeting the site that yielded the most acute symptom relief, with the occipital cortex and cerebellar vermis emerging as the preferred locations for most participants. In addition to reductions in vertigo intensity, dizziness, and balance disturbances, the authors reported a significant decrease in anxiety symptoms, with improvements persisting for up to six weeks post-treatment. Notably, no significant effects were observed on depressive symptoms.

In the second study, Yao et al. [20] investigated the effects of cerebellar TMS in patients with Alzheimer’s disease (AD), in light of converging evidence linking cerebellar dysfunctions to this condition (e.g., [97]). In their research, the authors demonstrated that four weeks of bilateral HF-rTMS over the cerebellum (with no cognitive training combined) significantly enhanced multiple cognitive domains in patients with AD. Specifically, active stimulation improved global cognition, episodic memory, executive function, verbal fluency, and visuospatial abilities, with gains evident immediately post-intervention and sustained at a 12-week follow-up. Importantly, these cognitive improvements were positively correlated with increased intrinsic functional connectivity between the cerebellar Crus II and key cortical regions implicated in cognition, including the dorsolateral prefrontal cortex, medial prefrontal cortex, and cingulate cortex. In contrast, the sham group showed no significant changes in cognitive performance or cerebello-cortical connectivity, underscoring the potential of cerebellar rTMS to facilitate neuroplasticity and strengthen functional integration within cerebello-cortical networks.

The third study, conducted by Wang et al. [34], investigated the effects of bilateral cerebellar cTBS targeting the dentate nucleus, delivered via a double-cone coil, in patients with drug-resistant epilepsy. After two weeks of treatment, participants receiving active stimulation exhibited a 34% reduction in seizure frequency within two months post-intervention compared to those in the sham group. Furthermore, cerebellar cTBS acutely suppressed interictal epileptiform discharges (IEDs) within 24 hours of stimulation, suggesting a potential immediate impact on electrophysiological hyperexcitability. However, no significant differences in IED frequency between the real and sham groups were observed at the one- and two-month follow-ups. In addition to its effects on seizure activity, cerebellar cTBS temporarily improved memory function at one month post-treatment, although this benefit was not maintained at the two-month evaluation. No significant changes were detected in global cognitive performance, mood, or quality of life at any assessed time points.

The limited body of research in this area underscores the disproportionate emphasis on motor disorders relative to cognitive and affective disturbances in cerebellar TMS studies. While preliminary, these findings collectively suggest that cerebellar TMS holds promise as a therapeutic strategy for non-motor neurological conditions. Nonetheless, further research is crucial to validate these initial observations, broaden the range of cerebellar TMS applications, clarify the underlying mechanisms, and establish the long-term efficacy and clinical utility of this approach.

### 4.4. Psychiatric Disorders

Among the studies reviewed, six investigated the effects of cerebellar TMS in psychiatric conditions, all focusing exclusively on schizophrenia. This focus reflects the growing evidence for altered functional connectivity both within the cerebellum and between cerebellar networks and cerebral regions involved in cognitive, affective, and sensory processing in patients with schizophrenia [98,99]. Moreover, reductions in grey matter volume in specific medial and lateral cerebellar lobules—particularly Crus I and II—have been positively correlated with cortical grey matter loss in fronto-temporo–parietal association areas and with clinical symptom severity in recent-onset schizophrenia, highlighting early cerebellar neuropathology in the disorder [100]. All six studies targeted the cerebellar vermis, motivated by early findings indicating that atrophy of this region is the most commonly reported structural cerebellar abnormality in schizophrenia (e.g., [101,102]). Consistently, reductions in negative symptoms—including blunted affect, decreased motivation, social withdrawal, and anhedonia—have been observed following ten sessions of iTBS [44,49,60] and HF-rTMS [45], with beneficial effects enduring from two weeks [45] up to 24 weeks post-intervention [60]. Importantly, cerebellar stimulation was associated with a reduction in gamma spectral power in the left fronto-temporal regions [44] and with increased functional connectivity between the cerebellum and right prefrontal regions [49,61]. These neural changes were positively correlated with reductions in symptom severity [44,49]. It has been suggested that the effects of cerebellar stimulation on distant cortical regions may result from an indirect inhibitory action on frontal and temporal cortices, triggered by excitation of the cerebellar vermis, and from a restoration of NMDA receptor function leading to enhanced long-term potentiation [44].

Despite these promising findings, results across studies remain inconsistent. For example, Basavaraju et al. [61], in line with previous research, reported increased resting-state functional connectivity between the cerebellar vermis and the right inferior frontal gyrus, right pallidum, and right frontal pole following real iTBS compared to sham stimulation. However, this enhanced connectivity did not correlate with changes in negative symptoms, which improved following both real and sham iTBS. The authors propose that the lack of association between changes in fronto-pallidal-cerebellar network connectivity and reductions in negative symptom severity may indicate that this network is not an optimal biological target for alleviating negative symptoms, but rather reflects a mechanism of action underlying vermal iTBS [61]. Alternatively, the observed improvements in negative symptoms, irrespective of stimulation condition, may suggest a substantial placebo effect, potentially contributing to the absence of differential efficacy. In a similar vein, Chauhan et al. [54] demonstrated that ten sessions of intensive iTBS (delivered twice daily) led to significant improvements across all assessed psychopathology domains; however, these benefits were not superior to those observed with sham stimulation. Notably, in this study, the cerebellar vermis was stimulated at a lower intensity (i.e., 80% of rMT) compared to other protocols (i.e., 100% rMT), which may have been insufficient to induce meaningful neurophysiological changes. Lastly, although some studies have documented improvements in mood and depressive symptoms alongside reductions in negative symptoms [44,45], other investigations have failed to replicate these findings [49,60]. However, the absence of research on other psychiatric conditions, such as depression and anxiety disorders, suggests a critical gap in the literature. Given the cerebellum’s established role in emotion regulation, further research into its neuromodulatory potential for psychiatric disorders is warranted.

Although these findings appear promising in the context of schizophrenia, it remains essential to carefully consider both patient characteristics and stimulation parameters, such as stimulation intensity and the specific cerebellar site targeted. To date, research has predominantly focused on the cerebellar vermis, leaving the potential benefits of stimulating other cerebellar regions largely unexplored. Furthermore, extending investigations beyond schizophrenia to include other psychiatric disorders—such as major depressive disorder, bipolar disorder, post-traumatic stress disorder, obsessive–compulsive disorder, and personality disorders—where cerebellar dysfunction has been implicated, represents a crucial avenue for future research. Broadening the scope in terms of clinical populations and stimulation protocols could significantly enhance our understanding of the cerebellum’s role in neuropsychiatric disease and its potential as a therapeutic target.

### 4.5. Technical Challenges and Recommendations

The reviewed studies exhibit high heterogeneity in their methodology, in terms of different sample characteristics, outcome measures, cerebellar target sites, as well as stimulation parameters, including coil type, intensity, and frequency. This discrepancy may explain some inconsistencies in the overall results. Still, it complicates the identification of the stimulation protocols that may be more effective in treating specific conditions and poses challenges for standardization. Nevertheless, despite this variability, the present review offers encouraging perspectives on the potential use of cerebellar TMS as a therapeutic tool in patients with various clinical conditions, particularly motor or psychiatric symptoms. Yet, we cannot exclude the possibility that publication bias may have influenced our conclusions, leading to an overestimation of the benefits of TMS due to a reduced tendency to disseminate null results. Therefore, the interpretations provided here must be taken with caution while awaiting both correctly powered and replication studies. 

The included studies employed various TMS protocols, with iTBS being the most frequently used. Improvements in motor, cognitive, and psychiatric symptoms were observed following iTBS and HF-rTMS, both of which are typically linked to excitatory effects on cortical excitability [103]. Notably, some studies also reported enhanced motor performance after cerebellar LF-rTMS and reduced seizure frequency in patients with drug-resistant epilepsy following cTBS—protocols generally considered inhibitory [103]. However, the notion that rTMS or other NIBS protocols inherently induce excitatory or inhibitory effects should be approached with caution, particularly when targeting non-motor areas. Neurophysiological and behavioral outcomes depend on multiple factors beyond simple changes in excitability, including the functional state of the stimulated region, interindividual anatomical variability, and differential responsiveness to stimulation [104]. Additionally, the effects of stimulation may also depend on the connectivity profile of the targeted region within broader functional networks, as different projections (e.g., excitatory vs. inhibitory) may modulate the outcome based on which network is engaged. This consideration is particularly relevant when stimulating the cerebellum, which has both excitatory and inhibitory projections to different brain regions (e.g., [105]). Indeed, given its entirely different cytoarchitecture compared to the neocortex, the cerebellar cell morphology, and the complex cerebellar folding, stimulation protocols are not predictive of the direction of the behavioral changes [106]. Moreover, even in motor regions, the classical frequency-based dichotomy does not always apply. For instance, although HF-rTMS over M1 has shown clinical benefits in PD [107,108], other studies have also reported positive effects using low-frequency protocols, highlighting the complexity of stimulation outcomes [108,109]. Within this context, further research is needed to clarify the mechanisms of cerebellar TMS, which remain only partially understood. Although cerebellar stimulation produces both local and network-level effects—such as cerebello-cortical and cortico-cortical synchronization [105,110,111]—the specific cerebellar structures most responsive to TMS remain unclear [103]. Modulatory effects may involve LTP/LTD-like processes at the level of inhibitory Purkinje cells [103,111], trans-synaptic activation via parallel or climbing fibers [103], or influences on granule cells and GABAergic interneurons [112]. Understanding these mechanisms will provide more reliable evidence and ultimately enhance the effectiveness of these interventions. 

Stimulation targets varied significantly, with most studies applying TMS to multiple cerebellar sites within the same session and reporting improvements in motor and cognitive functions. While this approach maximizes coverage, it leaves open the question of whether stimulating multiple sites is actually required, or if a single, well-targeted region would suffice. This issue is particularly relevant when considering the growing evidence supporting the existence of a complex functional specialization of the cerebellar contribution to motor and non-motor functions [113]. In the context of psychiatric applications, all reviewed studies targeted the cerebellar vermis, consistent with neuroimaging data highlighting its functional connectivity with the salience network [33,114,115], which is implicated in detecting and orienting attention toward emotionally salient stimuli [116]. However, recent evidence from our group [29] shows that while medial posterior cerebellar regions are engaged in lower-level socio-emotional processes, more lateral areas are recruited during higher-order social cognition, supporting a topographical organization of cerebellar contributions even within the socio-affective domain. On this point, it is worth noting that among the reviewed articles, only ten used individual MRI or estimated MRI, to localize the target regions, in all other cases, the regions of interest were localized by using craniometrics points, which provide a less precise localization. This limitation raises concerns about whether the observed therapeutic effects are due to specific stimulation of cerebellar circuits or broader, non-specific neuromodulatory effects. In light of this multifaceted view of cerebellar function, there is a need for research systematically exploring the effects of targeting distinct cerebellar subregions, more precise localization methods such as individual MRI, and computational modeling of the electric field distribution. If this issue is relevant when targeting all cerebral regions, it is even more important for cerebellar stimulation, considering the convoluted structure of cerebellar cortex, and particularly in conditions of increased atrophy or sulci width alterations. This will help in developing personalized interventions tailored to the patient’s specific physiological and clinical profile.

Regarding stimulation intensity, many studies have applied stimulation at 80% of the rMT and reported beneficial effects (but see [54]), suggesting that this intensity may be sufficient to modulate cerebellar activity. However, studies using higher intensities (e.g., 90%) have not consistently observed significant modulation. In comparison, those employing lower intensities (e.g., 50% of the maximum stimulator output) have also reported positive effects, complicating the identification of an optimal stimulation intensity. It is important to note, however, that the effectiveness of a given intensity in modulating cerebellar activity critically depends on the type of coil used (e.g., double-cone coil vs. figure-of-eight coil) [117,118]. Therefore, careful selection of both coil type and stimulation intensity is essential to align with the specific goals of the intervention. The majority of the reviewed studies employed a figure-of-eight coil, known for its capacity to deliver focal stimulation and commonly used to target relatively superficial brain regions—most likely at the level of the posterior lobe of the cerebellum. Nevertheless, several studies have reported improvements in motor functions associated with anterior cerebellar activation even when using this coil. Similar outcomes have also been observed with the double-cone coil, which is designed to reach deeper-lying structures. However, it is worth considering that, due to the anatomical distance between the scalp and the cerebellar motor representations in the anterior lobe, the motor effects observed with either coil type may have resulted from stimulation of more posterior regions of the cerebellum [118].

Regarding treatment duration, the studies included in this review employed a minimum of five stimulation sessions and reported mixed results. At least 10 to 12 daily sessions may be necessary to achieve more robust and consistent effects. An alternative to increase efficacy while avoiding a substantial extension of treatment duration is the use of accelerated cerebellar TMS protocols, which involve administering two TMS sessions per day. To date, a few studies have applied this protocol to the cerebellum and reported promising results [49,54,61,75,79,80,84,87]; however, further research is needed to better define the appropriate number of sessions. Moreover, while many studies have demonstrated short-term improvements (immediately following treatment), the long-term stability of these effects remains uncertain. Indeed, among the studies that explored the durability of stimulation effects, several documented improvements lasting 2 to 4 weeks after the intervention [45,52,64,70,73,82,84,86], with a few studies observing stable effects even 12 or 24 weeks after the intervention [20,46,60]. However, due to the heterogeneity in methodologies, follow-up durations, and outcome measures across studies, it is not yet possible to draw definitive conclusions regarding the long-term stability of the effects. Future research employing standardized follow-up periods and consistent assessment tools will be essential to better determine the durability of treatment benefits. 

### 4.6. Tolerability, Safety and Ethical Concerns

According to the available evidence, cerebellar TMS appears well-tolerated and safe, with no reports of moderate or severe adverse events. Reported side effects were minor and transient, including headache, posterior neck muscle contraction, nausea, vertigo, and fatigue. Based on the data reported in the analyzed studies, 22 patients experienced headaches, 15 reported posterior neck muscle contractions, 3 reported vertigo, 3 nausea, and 1 fatigue following real cerebellar TMS. However, it is important to note that data on minor side effects were not consistently reported across all studies, and detailed information regarding the intensity, duration, and frequency of these symptoms was rarely provided, underscoring the need for more systematic and standardized assessment of tolerability in future studies. Only a few participants discontinued their involvement, typically for reasons unrelated to the stimulation itself. Notably, two patients with schizophrenia undergoing antipsychotic treatment developed symptoms suggestive of mania or hypomania following real cerebellar iTBS; these symptoms resolved either spontaneously or with pharmacological management ([61]; for detailed case reports, see [119]). Given the growing interest in cerebellar rTMS for clinical applications, systematic monitoring and standardized reporting of both major and minor adverse events are essential. While the low incidence of acute side effects is encouraging, current evidence remains limited—particularly regarding long-term follow-up and outcomes in vulnerable populations. Future studies should prioritize the longitudinal assessment of safety, including the effects of cumulative exposure and the potential for delayed-onset reactions, to ensure the safe and responsible integration of cerebellar rTMS into clinical practice.

While cerebellar stimulation appears to be generally safe and well-tolerated, it may cause mild discomfort compared to non-cerebellar stimulation, primarily due to muscle contractions in the posterior neck region. This discomfort, although transient, could impact participant compliance and tolerability. To mitigate this issue, stabilizing the patient’s head with a chinrest—an approach successfully used in studies involving healthy individuals (e.g., [120])—could help minimize involuntary head movements and reduce muscular strain. Additionally, allowing brief pauses during stimulation sessions may further enhance comfort and adherence, ensuring optimal conditions for therapeutic application. Given the cerebellum’s greater depth relative to the scalp, many studies employ a double-cone coil to deliver stimulation, particularly when targeting anterior cerebellar regions. However, this approach raises the possibility of unintended stimulation of adjacent structures, such as the brainstem, which could lead to undesirable effects. To mitigate these risks, it is crucial to determine appropriate stimulation parameters based on simulations of the electric field distribution. These computational models can help optimize coil placement and stimulation intensity, ensuring that the intended cerebellar regions are effectively modulated while minimizing off-target activation. Future research should continue refining these methodologies to enhance the precision and safety of cerebellar neurostimulation in both research and clinical applications.

When applying TMS in individuals with neurological or psychiatric disorders, medication use is a critical consideration, as many patients take psychotropic or disease-modifying drugs. While earlier guidelines advised caution when stimulating individuals on medications that lower seizure threshold [121], recent evidence suggests that the risk of TMS-induced seizures is extremely low (<0.03%) and does not cause permanent harm [122]. However, caution remains necessary for populations with inherently higher seizure risk (e.g., stroke, traumatic brain injury) or those on seizure-lowering medications, requiring individualized risk–benefit assessments before modifying treatments. To ensure safety and optimize clinical decision-making, researchers should document concurrent drug use and other seizure risk factors (e.g., sleep deprivation, infection, alcohol consumption) while adhering to standardized procedures for reporting tolerability, side effects, and adverse events. Clinical oversight is crucial when enrolling vulnerable populations, as it involves balancing the risks and benefits of alternative treatments or no intervention. Additionally, ethical considerations surrounding informed consent must be addressed, with enhanced safeguards such as caregiver or proxy consent and adapted consent formats (e.g., simplified text, visual aids) to facilitate understanding in individuals with decisional impairments.

### 4.7. Future Directions

Our systematic review highlights a predominant emphasis on the application of cerebellar TMS for the treatment of motor disorders, with numerous studies reporting significant improvements in balance, gait, tremor, and ataxia severity. This trend aligns with the traditional view of the cerebellum as primarily involved in sensorimotor control, which has naturally directed early research toward its potential in motor rehabilitation. Nevertheless, today we can count on solid evidence linking the cerebellum to a broad range of cortical and subcortical networks involved in high-level cognitive functions, such as language, visuo-spatial abilities, executive functions, including working memory, attention, and inhibitory processes [3]. Yet, in the present review, only two studies have applied this approach to cognitive rehabilitation, representing an almost completely unexplored road for future studies. Indeed, grounded on evidence on healthy individuals supporting the possibility to modulate cognitive performance through cerebellar neurostimulation (for review and meta-analysis see [18,123]), the posterior cerebellum may represent a valid target for future trials aiming at boosting cognitive recovery following either acute injuries (e.g., stroke, TBI) or neurodegenerative diseases (e.g., AD, PD, frontotemporal dementia). 

In the psychiatric domain, studies have focused exclusively on patients with schizophrenia, reporting promising improvements in negative symptoms, including blunted affect, reduced motivation, anhedonia, and social withdrawal. Nevertheless, structural and functional alterations in cerebellar circuits have been observed in a range of psychiatric disorders—including mood disorders [124], eating disorders [125], post-traumatic stress disorder [126] and personality disorders [127]—suggesting the cerebellum may serve as a transdiagnostic biomarker of vulnerability [7]. These findings underscore the need for future research to broaden the application of cerebellar TMS to patients with cognitive impairment and psychiatric populations, with the aim of establishing it as a transdiagnostic therapeutic tool.

Beyond rTMS and TBS, more advanced protocols have emerged with the potential to modulate functional connectivity more precisely. One such technique is cortico-cortical paired associative stimulation (ccPAS), which involves delivering temporally coordinated TMS pulses to two distinct cortical sites via separate coils. The interstimulus interval determines whether effective connectivity between the regions is strengthened or weakened through spike-timing dependent plasticity mechanisms (see [128]). Among the studies reviewed, only one [65] implemented this protocol—targeting the cerebellum and motor cortex in post-stroke patients—and reported improvements in hand motor function. These findings point to ccPAS as a promising neuromodulatory approach for clinical populations with disrupted cortico-cerebellar connectivity.

In light of the growing complexity of TMS protocols, personalizing treatment parameters is becoming increasingly essential to enhance therapeutic efficacy. Individual variability in brain anatomy, connectivity, and responsiveness to stimulation can substantially influence outcomes, underscoring the need to tailor stimulation site, intensity, frequency, and timing based on functional or biomarker-based profiles. In this context, closed-loop stimulation paradigms—offering real-time adaptation of parameters based on neural or behavioral feedback—are emerging as a promising advancement, with initial applications integrating cerebellar TMS with techniques such as ccPAS, EEG, fMRI, or behavioral monitoring. These adaptive strategies may be particularly beneficial in disorders involving disrupted cerebello-cortical dynamics. However, despite encouraging preliminary evidence, the neurophysiological mechanisms underlying cerebellar TMS effects remain poorly understood, especially in clinical populations. Future research should combine TMS with neuroimaging and electrophysiological methods (e.g., fMRI, DTI) to clarify its impact on large-scale networks supporting motor, cognitive, and affective functions. Furthermore, although current findings support the safety and potential efficacy of cerebellar TMS, particularly in motor rehabilitation, there is a critical need for rigorously designed randomized controlled trials employing standardized protocols, robust methodological controls, and consistent outcome measures to validate and refine its clinical application.

## 5. Conclusions

Multiple sessions of cerebellar TMS represent a safe and promising neuromodulatory intervention, with growing evidence supporting their efficacy in improving motor symptoms across various neurological conditions and, to a lesser extent, in alleviating psychiatric symptoms such as those observed in schizophrenia. Although preliminary findings on cognitive outcomes are encouraging, further research is needed to clarify the therapeutic potential of cerebellar TMS in this domain. Future studies should aim to optimize stimulation protocols, enhance methodological rigor, and investigate the long-term effects and underlying neural mechanisms. These advancements could ultimately support the development of integrated and potentially transdiagnostic rehabilitative interventions targeting shared cerebello-cortical dysfunctions across diverse clinical populations.

## Figures and Tables

**Table 1:** Methodological features of TMS studies.

Author and Year	Sample Size, Mean Age (SD)	Diagnosis	Study Design	Sham-Controlled	Blinding	Follow-Up	Medications	Primary Outcome	Outcome Domain	Modulation Effect
Tikka et al., 2015 [44]	11, 24.63 (3.35)	Schizophrenia	Within-subject	No	Single-blind	No	Psychotropic Medication	PANSS, CDSS	Physiological/Socio-affective	Yes (+)
Garg et al., 2016 [45]	40, 32.40 (8.44)	Schizophrenia	Between-subject	Yes	Double-blind	Yes	Psychotropic Medication	PANSS	Socio-affective	Yes (+)
Bradnam et al., 2016 [46]	16, 51.95 (11.55)	Cervical Dystonia	Between-subject	Yes	Double-blind	Yes	Not Reported	TWSTRS	Motor	Yes (+)
Johkura et al., 2018 [47]	6, 53.5 (9.33)	Post-stroke Dizziness	Within-subject	No	Not reported	Yes	No Medication That Could Affect the Dizziness	VOR, DHI, Nystagmus	Motor	Yes (+)
Liu et al., 2018 [48]	9, 58.0 (7.0)	MSA	Within-subject	No	Not Reported	No	Dopaminergic Treatments	UMSARS, rs-fMRI	Motor	Yes (+)
Brady et al., 2019 [49]	11, 35.55 (10.50)	Schizophrenia	Between-subject	Yes	Double-blind	Yes	Not Reported	PANSS, rs-fMRI	Physiological/Socio-affective	Yes (+)
Koch et al., 2019 [50]	34, 64 (11.3)	Post-stroke Hemiparesis	Between-subject	Yes	Double-blind	No	Not Reported	BBS	Motor	Yes (+)
Cha et al., 2019 [51]	26, 51.3 (12.4)	Mal de Débarquement Syndrome	Within-subject	No	Single-blind	Yes	Not reported	DHI, MBRS, HADS	Motor	Yes (+)
Manor et al., 2019 [52]	20, 51 (6,5)	SCA	Between-subject	Yes	Double-blind	Yes	Not reported	SARA	Motor	Yes (+)
Shin et al., 2019 [53]	21, 67 (9.25)	Essential tremor	Between-subject	Yes	Single-blind	Yes	Yes, Not reported	TRS, MEPs	Motor	No
Chauhan et al., 2020 [54]	30, 40.05 (8,54)	Schizophrenia	Between-subject	Yes	Double-blind	Yes	Yes, Psychotropic Medication	PANSS	Socio-affective	No
Tramontano et al., 2020 [55]	20, 51.75 (7.37)	Multiple Sclerosis	Between-subject	Yes	Double-blind	No	Not Reported	TBG	Motor	Yes (+)
Olfati et al., 2020 [56]	23, 60 (15.38)	Essential Tremors	Cross-over	Yes	Double-blind	Yes	Not Reported	FTM	Motor	No
França et al., 2020 [57]	24, 49 (13,8)	Cerebellar Ataxia (MSA-c, Post-lesion Ataxia, SCA3)	Cross-over	Yes	Double-blind	Yes	Yes, Not Reported	SARA	Motor	Yes (+)
Song et al., 2020 [58]	20, 51.1 (9.2)	MSA-C	Between-subject	Yes	Double-blind	No	Not reported	SARA, EEG activity	Physiological/Motor	Yes (+)
Liao et al., 2020 [59]	30, 54.2 (8.66)	Post-stroke	Between-subject	Yes	Double-blind	Yes	Not Reported	BBS	Motor	Yes (+)
Zhu et al., 2021 [60]	64, 35.25 (6.63)	Schizophrenia	Between-subject	Yes	Double-blind	Yes	Antipsychotic Medication	PANSS	Socio-affective	Yes (+)
Basavaraju et al., 2021 [61]	30, 32.67 (8.98)	Schizophrenia	Between-subject	Yes	Double-blind	Yes	Antipsychotic Medication	SANS, rs-fMRI	Physiological/Socio-affective	Yes (+)
Chen et al., 2021 [62]	32, 54.41 (8.62)	Post-stroke Upper Limb Spasticity	Between-subject	Yes	Double-blind	No	Not Reported	MAS, MTS, SWV	Motor	Yes (+)
Li et al., 2021 [63]	90, 56.5 (7.96)	Post-stroke Hemiplegia	Between-subject	No	Not Reported	No	Not Reported	MAS, FMA, BI	Motor	Yes (+)
Yao et al., 2022 [20]	27, 65.74 (7.37)	Alzheimer’s Disease	Between-subject	Yes	Double-blind	Yes	Acetylcholinesterase Inhibitors	MMSE, MoCA, CDR, ADAS, Cog, RAVLT, CDT, BNT, VFT, TMT, SDMT, DST, HAMD, HAMA, PSQI, ADL	Cognitive	Yes (+)
Rao et al., 2022 [64]	70, 64.66 (10.89)	Post-stroke Dysphagia	Between-subject	Yes	Double-blind	Yes	Benzodiazepine and Antidepressants	FEDSS	Motor	Yes (+)
Rosso et al., 2022 [65]	27, 61.5 (12.5)	Post-stroke	Between-subject	Yes	Double-blind	Yes	Not Reported	JTT, Grip Strength	Motor	Yes (+)
Chen et al., 2022 [66]	18, 39.78 (9.23)	SCA3	Between-subject	Yes	Double-blind	No	Not Reported	ICARS	Motor	Yes (+)
Dong et al., 2022 [67]	36, 53.8 (10.13)	Post-stroke Dysphagia	Between-subject	Yes	Not Reported	No	Not Reported	PAS, FDS	Motor	Yes (+)
Sanna et al., 2022 [68]	6, 50.33 (4.68)	SCA38	Cross-over	Yes	Double-blind	No	Not Reported	MICARS, MEPs, DNA analysis	Motor	Yes (+)
Lv et al., 2023 [69]	38, 55.45 (6.86)	Essential Tremors	Between-subject	No	Single-blind	Yes	Yes, Propranolol	FTM	Motor	No
Dai et al., 2023 [70]	42, 60.09 (2.84)	Post-stroke	Between-subject	Yes	Single-blind	Yes	Not Reported	FOIS	Motor	Yes (+)
Shi et al., 2023 [71]	120, 42.29 (9.49)	SCA3	Between-subject	Yes	Single-blind	Yes	Yes, Neuroprotective Medications	SARA, ICARS	Motor	Yes (+)
Sikandar et al., 2023 [72]	44, 39.42 (9.67)	SCA3	Between-subject	Yes	Double-blind	No	Yes, Stop Ataxia Medications	ICARS	Motor	Yes (+)
Zhong et al., 2023 [73]	84, 63.21 (10.58)	Post-stroke Dysphagia	Between-subject	Yes	Double-blind	Yes	Not Reported	FEDSS	Motor	Yes (+)
Zhu et al., 2024 [74]	36, 60.5 (8.01)	Post-stroke Hemiplegia	Between-subject	Yes	Double-blind	No	Not Reported	BBS	Motor	Yes (+)
Grobe-Einsler et al., 2024 [75]	35, 68.24 (10.04)	Parkinson’s Disease	Between-subject	Yes	Double-blind	Yes	Levodopa	Dynamic Posturography	Motor	Yes (+)
Yassine et al., 2024 [76]	39, 39.9 (6.1)	Relapsing-remitting Multiple Sclerosis	Phase 1: Between-Subject Phase 2: Within-subject	Yes	Single-blind	Yes	Disease-modifying Medications	10MWT, TUG, BBS, DTI	Motor	Yes (+)
Liu et al., 2024 [77]	22, 42.09 (10.9)	SCA3	Between-subject	Yes	Double-blind	No	Not Reported	ICARS, rs-fMRI	Physiological/Motor	Yes (+)
Zhou et al., 2024 [78]	16, 39.72 (10.14)	SCA3	Between-subject	No	Double-blind	No	Yes, Not Reported	SARA, ICARS	Motor	Yes (+)
Grobe-Einsler et al., 2024 [79]	33, 48.5 (13.8)	SCA	Between-subject	Yes	Double-blind	Yes	Yes, Not Reported	SARA	Motor	Yes (+)
Grobe-Einsler et al., 2024 [80]	8, 65.1 (6.1)	CANVAS	Between-subject	Yes	Double-blind	No	Yes, Not Reported	SARA	Motor	Yes (+)
He et al., 2024 [81]	20, 64.7 (8.40)	Essential Tremor	Within-subject	No	Not Reported	No	Yes, Anti-tremor Medication discontinued	TETRAS, rs-EEG	Physiological/Motor	Yes (+)
Grobe-Einsler et al., 2024 [82]	36, 69.5 (10.0)	Parkinson’s disease	Between-subject	Yes	Double-blind	Yes	Yes, Antiparkinsonian Medication	UPDRSIII	Motor	Yes (+)
El-Adawy et al., 2024 [83]	45, 38.77 (4.8)	Essential Tremor	Between-subject	Yes	Single-blind	No	Yes, Not Reported	FTM	Motor	Yes (+)
Liao et al., 2024 [84]	36, 57.5 (12.51)	Post-stroke Lower Limb Dysfunction	Between-subject	Yes	Double-blind	Yes	Not Reported	BBS	Motor	Yes (+)
Zhang et al., 2024 [85]	24, 56.99 (7.21)	Post-stroke	Between-subject	No	Not reported	No	No Psychotropic Medications	EEG activity, BBS, HMDS, HAMA, MADRS, IDSSR, MMSE, MoCA, WMT, BNT	Physiological/Motor, Cognitive, affective	Yes (+)
Li et al., 2025 [86]	26, 66.81 (4.08)	MSA-C	Between-subject	Yes	Double-blind	Yes	Yes, Not Reported	SARA	Motor and Affective	Yes (+)
Wang et al., 2025 [34]	38, 31.0 (11.64)	Drug-resistant Epilepsy	Cross-over	Yes	Double-blind	Yes	Antiseizure Medications	% of Seizure Reduction	Epilepsy	Yes (+)
Ye et al., 2025 [87]	20, 50.60 (4.72)	Post-Stroke Unilateral Neglect	Between-subject	Yes	Not Reported	No	Not Reported	Line Cancellation Task, Star Cancellation Task, Line Bisection Task, rs-EEG	Cognition	Yes (+)

PANSS—Positive and Negative Syndrome Scale; CDSS—Calgary Depression Scale for Schizophrenia; TWSTRS—Toronto Western Spasmodic Torticollis Rating Scale; VOR—Vestibulo-Ocular Reflex; DHI—Dizziness Handicap Inventory; UMSARS—Unified Multiple System Atrophy Rating Scale; rs-fMRI—Resting-State Functional Magnetic Resonance Imaging; BBS—Berg Balance Scale; MBRS—Movement Disorders Rating Scale; HADS—Hospital Anxiety and Depression Scale; SARA—Scale for the Assessment and Rating of Ataxia; TRS—Tremor Rating Scale; MEPs—Motor Evoked Potentials; TBG—Total Body Gait; FTM—Fahn–Tolosa–Marin Tremor Rating Scale; SANS—Scale for the Assessment of Negative Symptoms; MAS—Modified Ashworth Scale; MTS—Modified Tardieu Scale; SWV—Shear Wave Velocity; FMA—Fugl–Meyer Assessment; BI—Barthel Index; MMSE—Mini-Mental State Examination; MoCA—Montreal Cognitive Assessment; CDR—Clinical Dementia Rating; ADAS-Cog—Alzheimer’s Disease Assessment Scale-Cognitive Subscale; RAVLT—Rey Auditory Verbal Learning Test; CDT—Clock Drawing Test; BNT—Boston Naming Test; VFT—Verbal Fluency Test; TMT—Trail Making Test; SDMT—Symbol Digit Modalities Test; DST—Digit Span Test; HAMD—Hamilton Depression Rating Scale; HAMA—Hamilton Anxiety Rating Scale; PSQI—Pittsburgh Sleep Quality Index; ADL—Activities of Daily Living; FEDSS—Functional Eating and Drinking Scale; JTT—Jebsen–Taylor Hand Function Test; ICARS—International Cooperative Ataxia Rating Scale; PAS—Postural Assessment Scale; FDS—Functional Disability Scale; MICARS—Modified International Cooperative Ataxia Rating Scale; FOIS—Functional Oral Intake Scale; 10MWT—10-Meter Walk Test; TUG—Timed Up and Go Test; DTI—Diffusion Tensor Imaging; TETRAS—Tremor Research Group Essential Tremor Rating Assessment Scale; rs-EEG—Resting-State Electroencephalography; UPDRS III—Unified Parkinson’s Disease Rating Scale, Part III; HMDS—Huntington’s Disease Rating Scale; MADRS—Montgomery–Åsberg Depression Rating Scale; IDSSR—Inventory of Depressive Symptomatology-Self Report; WMT—Word Memory Test.

**Table 2:** Technical details of the TMS protocols.

Author and Year	TMS Protocol	Coil (Diameter)	Target Site (s)	MRI-guided	Frequency and Intensity	Training	N Sessions and Duration	Safety and Tolerability	Adverse Events
Tikka et al., 2015 [44]	iTBS	Double-cone	Vermis	No	5, 6, and 7 Hz 100% MT	No	10 (2 weeks)	Not Reported	Not Reported
Garg et al., 2016 [45]	HF-rTMS	Double-cone	Vermis	No	5, 6, and 7 Hz 100% MT	No	10 (2 weeks)	Yes	Headache and Sleepiness
Bradnam et al., 2016 [46]	iTBS	Figure 8 (70 mm)	L Cerebellum R Cerebellum	No	50 Hz bursts at 5 Hz 80% MT	Active Exercise Training	10 (2 weeks)	Yes	None
Johkura et al., 2018 [47]	iTBS	Double-cone	Vermis	No	50 Hz Bursts at 5 Hz 80% MT	No	5 (5 days)	Yes	Discomfort from Posterior Neck Muscle Contraction
Liu et al., 2018 [48]	HF-rTMS	Circular (50 mm)	L M1 R M1 L Cerebellum R Cerebellum	No	5 Hz 100% MT	No	5 (5 days)	Yes	None
Brady et al., 2019 [49]	iTBS	Figure 8 (70 mm)	Vermis	Yes	50 Hz Bursts at 5 Hz 100% MT	No	10 (5 days)	Not Reported	Not Reported
Koch et al., 2019 [50]	iTBS	Figure 8 (70 mm)	Contralesional Lateral Cerebellum	Yes	50 Hz bursts at 5 Hz 80% MT	Physiotherapy	15 (3 weeks)	Yes	None
Cha et al., 2019 [51]	cTBS	Double-cone	Occipital Cortex Vermis L Cerebellum R Cerebellum	Yes	50 Hz Bursts at 5 Hz Maximum Tolerated Level	No	10-12 (5 days)	Yes	Neck Muscle Contraction
Manor et al., 2019 [52]	LF-rTMS	Circular (140 mm)	Vermis, L Cerebellum R Cerebellum	Yes	1 Hz 100% MSO	No	20 (4 weeks)	Yes	None
Shin et al., 2019 [53]	LF-rTMS	Figure 8 (70 mm)	L Cerebellum R Cerebellum	No	1 Hz 90% MT	No	5 (5 days)	Yes	None
Chauhan et al., 2020 [54]	iTBS	Figure 8 (70 mm)	Vermis	No	50 Hz bursts at 5 Hz 80% MT	No	10 (5 days)	Yes	Headache
Tramontano et al., 2020 [55]	iTBS	Figure 8 (70 mm)	L Cerebellum R Cerebellum	Yes	50 Hz Bursts at 5 Hz 80% MT	Gaze Stability and Postural Stability	10 (2 weeks)	Not Reported	Not Reported
Olfati et al., 2020 [56]	LF-rTMS	Figure 8 (100 mm)	L Cerebellum R Cerebellum	No	1 Hz 90% MT	No	5 (5 days)	Yes	Headache and Local Pain
França et al., 2020 [57]	LF-rTMS	Double-cone	Contralesional Lateral Cerebellum	Yes	1 Hz 90% MT	No	5 (5 days)	Yes	Headache and Short-lasting Worsening of Left Leg Pain
Song et al., 2020 [58]	iTBS	Figure 8 (70 mm)	L Cerebellum R Cerebellum	No	50 Hz Bursts at 5 Hz 80% MT	No	10 (2 weeks)	Yes	None
Liao et al., 2020 [59]	iTBS	Figure 8 (70 mm)	Contralesional Lateral Cerebellum	No	50 Hz Bursts at 5 Hz 80% MT	Physiotherapy	10 (2 weeks)	Yes	None
Zhu et al., 2021 [60]	iTBS	Figure 8 (Not reported)	Vermis	No	50 Hz Bursts at 5 Hz 100% MT	No	10 (2 weeks)	Not Reported	Not Reported
Basavaraju et al., 2021 [61]	iTBS	Figure 8 (70 mm)	Vermis	Yes	50 Hz Bursts at 5 Hz 100% MT	No	10 (5 days)	Yes	Neck Muscle Contraction and Hypomania
Chen et al., 2021 [62]	iTBS	Figure 8 (70 mm)	Ipsilesional Lateral Cerebellum	No	50 Hz Bursts at 5 Hz 80% MT	Physical Therapy	10 (2 weeks)	Yes	None
Li et al., 2021 [63]	LF-rTMS cTBS LF-rTMS + cTBS	Circular (Not Reported)	M1 R Cerebellum	No	1 Hz 50 Hz Bursts at 5 Hz 80% MT	Rehabilitative Training and Acupuncture Therapy	24 (4 weeks)	Not Reported	Not Reported
Yao et al., 2022 [20]	HF-rTMS	Figure 8 (70 mm)	L Cerebellum R Cerebellum	No	5 Hz 90% MT	No	20 (4 weeks)	Yes	None
Rao et al., 2022 [64]	iTBS	Figure 8 (90 mm)	L Cerebellum R Cerebellum	No	50 Hz Bursts at 5 Hz 100% MT	Dysphagia Therapy	10 (2 weeks)	Yes	None
Rosso et al., 2022 [65]	Cerebellum-M1 PAS	Double-cone	Contralesional Lateral Cerebellum	No	120 pairs of pulses at 0.2 Hz 90% MT	Physical Therapy	5 (1 week)	Yes	Headache, Reflex Syncope and Discomfort
Chen et al., 2022 [66]	LF-rTMS	Figure 8 (Not Reported)	L Cerebellum R Cerebellum	No	1 Hz Not Reported	No	15 (15 days)	Yes	None
Dong et al., 2022 [67]	HF-rTMS	Circular (70 mm)	L Cerebellum R Cerebellum	No	10 Hz 80% MT	Swallowing Rehabilitation	10 (2 weeks)	Not Reported	Not Reported
Sanna et al., 2022 [68]	iTBS	Figure 8 (70 mm)	L Cerebellum R Cerebellum	No	50 Hz Bursts at 5 Hz 80% MT	No	10 (2 weeks)	Not Reported	Not Reported
Lv et al., 2023 [69]	LF-rTMS	Figure 8 (70 mm)	L Cerebellum R Cerebellum	No	1 Hz 90% MT	No	10 (10 days)	Yes	None
Dai et al., 2023 [70]	iTBS	Double-cone	L Cerebellum R Cerebellum	No	50 Hz Bursts at 5 Hz 90% MT	No	10 (2 weeks)	Yes	Twitching Feeling
Shi et al., 2023 [71]	LF-rTMS iTBS	Figure 8 (70 mm)	L Cerebellum R Cerebellum	No	1 Hz 50 Hz Bursts at 5 Hz 100% MT	No	10 (2 weeks)	Yes	None
Sikandar et al., 2023 [72]	LF-rTMS	Circular (140 mm)	L Cerebellum R Cerebellum	No	1 Hz 100% MT	No	15 (15 days)	Yes	Nausea
Zhong et al., 2023 [73]	HF-rTMS	Figure 8 (90 mm)	L Cerebellum R Cerebellum	No	10 Hz 80% MT	Swallowing Rehabilitation	10 (10 days)	Yes	Neck Twitching
Zhu et al., 2024 [74]	iTBS	Figure 8 (Not Reported)	Ipsilesional Lateral Cerebellum	No	50 Hz Bursts at 5 Hz 80% MT	Physical Therapy	10 (2 weeks)	Yes	None
Grobe-Einsler et al., 2024 [75]	iTBS	Figure 8 (70 mm)	Vermis L Cerebellum R Cerebellum	No	48 Hz bursts at 5 Hz 50% MSO	No	10 (5 days)	Yes	Headache, Fatigue and Head/Neck Muscle Tension
Yassine et al., 2024 [76]	iTBS	Figure 8 (70 mm)	Vermis L Cerebellum R Cerebellum	No	50 Hz Bursts at 5 Hz 80% MT	No	Phase I: 12 (4 Weeks) Phase II: 48 (1 Year)	Yes	Nausea, Vomiting, Headache and Neck Stiffness.
Liu et al., 2024 [77]	LF-rTMS	Figure 8 (Not Reported)	L Cerebellum R Cerebellum	No	1 Hz 100% MT	No	15 (2 weeks)	Yes	None
Zhou et al., 2024 [78]	HF-rTMS	Double-cone	Vermis L Cerebellum R Cerebellum	No	10 Hz 100% MT	No	10 (2 weeks)	Yes	Not Reported
Grobe-Einsler et al., 2024 [79]	iTBS	Figure 8 (70 mm)	Vermis L Cerebellum R Cerebellum	Yes	48 Hz Bursts at 5 Hz 50% MSO	Physiotherapy	15 (5 days)	Yes	Headache
Grobe-Einsler et al., 2024 [80]	iTBS	Figure 8 (70 mm)	Vermis L Cerebellum R Cerebellum	Yes	48 Hz Bursts at 5 Hz 50% MSO	Speech and Physiotherapy	15 (5 days)	Yes	Local Pain over the Stimulation Area
He et al., 2024 [81]	LF-rTMS	Not Reported	L Cerebellum R Cerebellum	No	1 Hz 90% MT	No	20 (4 weeks)	Not Reported	Not Reported
Grobe-Einsler, et al., 2024 [82]	iTBS	Figure 8 (70 mm)	Vermis L Cerebellum R Cerebellum	No	48 Hz Bursts at 5 Hz 50% MSO	No	15 (5 days)	Yes	Headache and Subjective Worsening of Gait Disturbance
El-Adawy et al., 2024 [83]	LF-rTMS	Figure 8 (100 mm)	L Cerebellum R Cerebellum	No	1 Hz 90% MT	No	12 (4 weeks)	Not Reported	Not Reported
Liao et al., 2024 [84]	iTBS	Figure 8 (70 mm)	Contralesional Lateral Cerebellum	No	50 Hz Bursts at 5 Hz 80% MT	No	15 (5 days)	Yes	Vertigo
Zhang et al., 2024 [85]	iTBS	Double-cone	Contralesional Lateral Cerebellum	No	50 Hz Bursts at 5 Hz 80% MT	Routine Rehabilitation	10 (2 weeks)	Not Reported	Not Reported
Li et al., 2025 [86]	iTBS	Figure 8 (70 mm)	L Cerebellum R Cerebellum	No	50 Hz Bursts at 5 Hz 80% MT	No	10 (2 weeks)	Yes	None
Wang et al., 2025 [34]	cTBS	Double-cone	L Cerebellum R Cerebellum	Yes	50 Hz Bursts at 5 Hz 80% MT	No	10 (2 weeks)	Yes	Headache, Tinnitus and Dizziness
Ye et al., 2025 [87]	iTBS	Figure 8 (92 mm)	L Cerebellum	No	50 Hz Bursts at 5 Hz 90% MT	Physical Therapy	20 (10 days)	Not Reported	Not Reported

LF: Low-frequency; HF: High-frequency; L: left; R: right; MT: motor threshold; MSO: maximum stimulator output.

**Table 3:** Results of the methodological assessment of non-RCT studies with pre-post design and no control group according to the NHLBI Quality assessment tool (National Heart, Lung, and Blood Institute, 2014).

NHLBI Quality Assessment Tool	Tikka et al., 2015 [44]	Johkura et al., 2018 [47]	Liu et al., 2018 [48]	Cha et al., 2019 [51]	He et al., 2024 [81]
*1. Was the study question or objective clearly stated?*	Y	Y	Y	Y	Y
*2. Were eligibility/selection criteria for the study population prespecified and clearly described?*	Y	Y	Y	Y	Y
*3. Were the participants in the study representative of those who would be eligible for the test/service/intervention in the general or clinical population of interest?*	Y	Y	Y	Y	Y
*4. Were all eligible participants that met the prespecified entry criteria enrolled?*	Y	Y	Y	Y	Y
*5. Was the sample size sufficiently large to provide confidence in the findings?*	N	N	N	N	Y
*6. Was the test/service/intervention clearly described and delivered consistently across the study population?*	Y	Y	Y	Y	Y
*7. Were the outcome measures prespecified, clearly defined, valid, reliable, and assessed consistently across all study participants?*	Y	Y	Y	Y	Y
*8. Were the people assessing the outcomes blinded to the participants’ exposures/interventions?*	Y	N	N	NR	NR
*9. Was the loss to follow-up after baseline 20% or less? Were those lost to follow-up accounted for in the analysis?*	N	N	Y	Y	NR
*10. Did the statistical methods examine changes in outcome measures from before to after the intervention? Were statistical tests done that provided p values for the pre-to-post changes?*	Y	Y	Y	Y	Y
*11. Were outcome measures of interest taken multiple times before the intervention and multiple times after the intervention (i.e., did they use an interrupted time-series design)?*	N	N	N	N	N
*12. If the intervention was conducted at a group level (e.g., a whole hospital, a community, etc.) did the statistical analysis take into account the use of individual-level data to determine effects at the group level?*	NA	NA	NA	NA	NA
**Overall Bias**	**Fair**	**Poor**	**Fair**	**Fair**	**Good**

## Data Availability

This systematic review has been registered in PROSPERO (registration number: CRD420251067308). The data used to support the findings of this study are available from the corresponding author upon request.
